# Dynamic cross-regulation of antigen-specific effector and regulatory T cell subpopulations and microglia in brain autoimmunity

**DOI:** 10.1186/1752-0509-7-34

**Published:** 2013-04-26

**Authors:** Sara Martinez-Pasamar, Elena Abad, Beatriz Moreno, Nieves Velez de Mendizabal, Ivan Martinez-Forero, Jordi Garcia-Ojalvo, Pablo Villoslada

**Affiliations:** 1 Center of Neuroimmunology, Institute of Biomedical Research August Pi Sunyer (IDIBAPS), Hospital Clinic of Barcelona, Barcelona, Spain; 2 Department of Experimental and Health Sciences, Universitat Pompeu Fabra, Barcelona, Spain; 3 School of Medicine and Indiana Clinical and Translational Sciences Institute, Indiana University, Indianapolis, IN, USA; 4 Hospital Pablo Tobón Uribe, Medellin, Colombia

**Keywords:** T cells, Effector, Regulatory, B cells, Dynamics, Autoimmunity, Multiple sclerosis, Systems biology, Immunotherapy, Anti-CD20

## Abstract

**Background:**

Multiple Sclerosis (MS) is considered a T-cell-mediated autoimmune disease with a prototypical oscillatory behavior, as evidenced by the presence of clinical relapses. Understanding the dynamics of immune cells governing the course of MS, therefore, has many implications for immunotherapy. Here, we used flow cytometry to analyze the time-dependent behavior of antigen-specific effector (T_eff_) and regulatory (T_reg_) T cells and microglia in mice model of MS, Experimental Autoimmune Encephalomyelitis (EAE), and compared the observations with a mathematical cross-regulation model of T-cell dynamics in autoimmune disease.

**Results:**

We found that T_eff_ and T_reg_ cells specific to myelin olygodendrocyte glycoprotein (MOG) developed coupled oscillatory dynamics with a 4- to 5-day period and decreasing amplitude that was always higher for the T_eff_ populations, in agreement with the mathematical model. Microglia activation followed the oscillations of MOG-specific T_eff_ cells in the secondary lymphoid organs, but they were activated before MOG-specific T-cell peaks in the CNS. Finally, we assessed the role of B-cell depletion induced by anti-CD20 therapy in the dynamics of T cells in an EAE model with more severe disease after therapy. We observed that B-cell depletion decreases T_eff_ expansion, although its oscillatory behavior persists. However, the effect of B cell depletion was more significant in the T_reg_ population within the CNS, which matched with activation of microglia and worsening of the disease. Mathematical modeling of T-cell cross-regulation after anti-CD20 therapy suggests that B-cell depletion may influence the dynamics of T cells by fine-tuning their activation.

**Conclusions:**

The oscillatory dynamics of T-cells have an intrinsic origin in the physiological regulation of the adaptive immune response, which influences both disease phenotype and response to immunotherapy.

## Background

Relapsing-remitting oscillatory behavior is a hallmark of autoimmune diseases such as Multiple Sclerosis (MS) [1,2]. At the clinical level, the presence of relapses defines the subtypes of MS. The clinical relapse rate is about 0.5-1 per year, with around a month of duration and self-resolution. At the pathological level, a relapse is the result of an acute inflammatory process within the central nervous system (CNS), which produces demyelination and axonal damage and impairs neural conduction, leading to clinical symptoms. Acute inflammatory lesions are revealed by magnetic resonance imaging (MRI) as contrast-enhancing lesions. The number of clinically defined relapses represents only one in every 5–10 contrast-enhancing lesions, as has been shown by MRI studies [3], because many brain regions are silent and brain plasticity copes tissue damage. It is not known what triggers relapse onset, although one third of the relapses are preceded by common infections or stressful events [4,5]. Understanding the biological basis of relapses in MS may have implications for immunotherapy.

In autoimmune diseases, the balance between the pro-inflammatory response and regulatory factors seems to be disrupted. As such, antigen specific effector T cells (T_eff_) are over-activated [6], whereas the function of regulatory lymphocytes (T_reg_) is altered [7-9], indicating impairment of peripheral tolerance. It could be expected that this impaired immune response would produce a chronic inflammatory process leading a progressive clinical course. However, in MS and other autoimmune diseases the predominant behavior is fluctuating, with periods of T-cell activation and tissue damage followed by deactivation and repair. Although this behavior could be partially accounted for by external triggers (e.g. viral infections, stress, changes in light exposure and vitamin D levels), its robustness among pathologies, resilience in front of immunotherapies and the lack of closely related triggers suggest that this may not be the only explanation. We have postulated that the oscillatory behavior of the autoimmune response arises from an underlying periodic dynamics that is intrinsic to the design of the immune system, with several control mechanisms providing negative feedbacks that tightly control T-cell activation [10]. Mathematical models predict that cross-regulation of T_eff_ and T_reg_ cells generates a stable oscillatory dynamic of both populations that maintains homeostasis in health, but which also promotes relapsing-remitting flares under autoimmune conditions [10-12]. The generation of an autoimmune response seems to be based in the presence of susceptibility factors such as genetic polymorphisms, levels of modulators of the immune response such as vitamin D, or previous infections that would change the thresholds of T-cell activation and differentiation, defining the autoimmune regime. However, the presence of environmental factors (e.g. vitamin D levels, acute infections or stress) or genetic polymorphism have not been convincingly associated with this oscillatory behavior, supporting the concept that such oscillations are inherent to the organization of the adaptive immune system.

B-cell depletion has recently emerged as a powerful immunotherapy for treating autoimmune diseases such as Rheumatoid Arthritis (RA), Lupus or MS [13,14]. Although originally conceived for targeting the role of antibodies in the pathogenesis of autoimmune diseases, the quick and profound response observed in clinical trials in patients with MS and RA suggest that instead of decreasing antibody production, B-cell depletion may work by modulating the pro-inflammatory environment created by B-cells, the antigen presentation function they provide to T-cells, or the function of regulatory B-cells [15,16]. Moreover, anti-CD20 therapy ameliorates the condition of the animal model of MS, Experimental Autoimmune Encephalitis (EAE), preventing the pro-inflammatory environment (Th1/Th17 responses) promoted by B-cells [17]. However, in the EAE model in which B cells do not play an encepalithogenic role, B-cell depletion decreases T_reg_ and regulatory B-cells, worsening the disease course [17]. Understanding the effects of B-cell depletion in T-cell activation would improve our understanding of the mechanisms governing peripheral tolerance and its role in autoimmune diseases.

Here we validate at the experimental level the hypothesis that the relapsing-remitting behavior of autoimmune diseases is highly dependent on the cross-regulation of the lymphocyte populations, demonstrating that the underlying oscillatory behavior is intrinsic to the design of the immune system. We have analyzed the dynamics of Myelin Oligodrendrocyte (MOG)-specific T_eff_ and T_reg_ cells in secondary lymphoid organs (spleen) and in the target tissue (CNS), as well as the activation of microglia in the animal model of MS, the Experimental Autoimmune Encephalitis (EAE). Moreover, we used our experimental and mathematical model to obtain insights into the role of anti-CD20 therapy in the regulation of T-cell dynamics and the generation of autoimmune response.

## Results

### Antigen specific T cell subpopulations and microglia display coupled and interlinked oscillatory dynamics during the autoimmune attack in the brain

**Figure 1:**
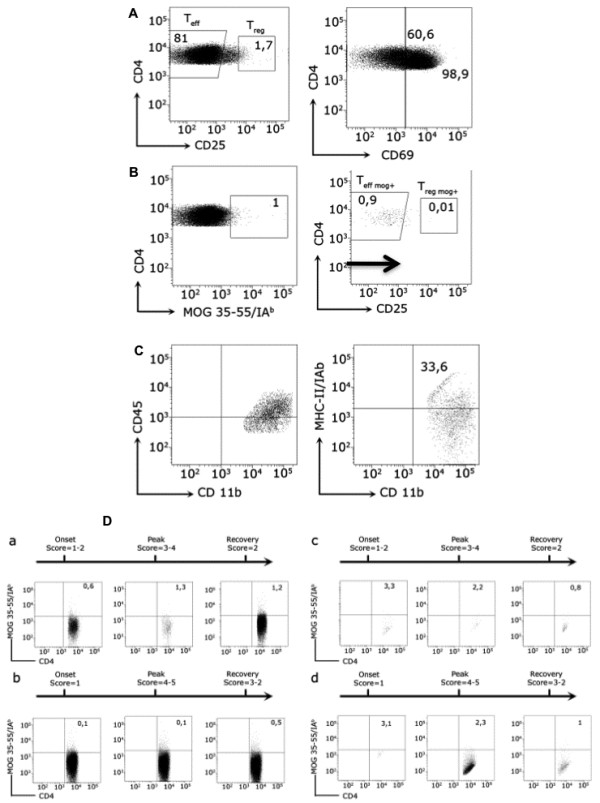
**MOG-specific T cells and microglia subpopulations analyzed by flow cytometry in mice with EAE.** Leukocytes were obtained from the spleen and the CNS from MOG_35-55_ immunized C57BL/6 mice at different stages of EAE as indicated. **A**) T_eff_ and T_reg_ lymphocytes are shown. Numbers indicate the percentage of cells within the CD4+ cell gate. The histogram on the right shows the expression of CD69 on the surface of T_eff_ and T_reg_ cell subsets in the spleen. Numbers indicate the percentage of CD69+ cells. **B**) CD4+ cells stained with MOG_35-55_/IA^b^ tetramer. MOG-specific T_eff_ and T_reg_ cells are shown. **C**) Dot-plots showing CD45^+^CD11b^+^MCH-II(IA^b^)^+^ microglia population. **D**) Antigen-specific T_eff_ and T_reg_ oscillate over the course of EAE. Numbers indicate the percentage within the CD4+ cell gate. MOG-specific T cells from spleen (**a**, **b**) and CNS (**c**, **d**) in the onset, peak and recovery stages of EAE are shown. Animals were not treated (**a**, **c**) or treated with anti-CD20 antibody before immunization (**b**, **d**).

**Figure 2:**
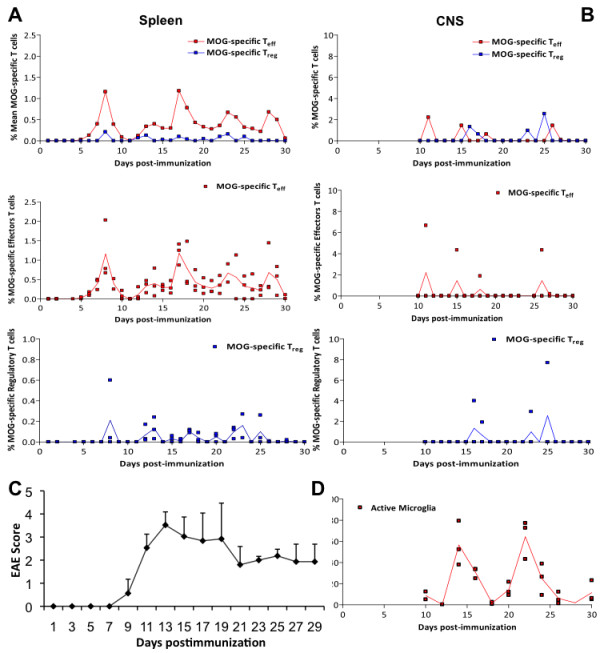
**Dynamics of activated MOG-T**_**eff**_**, MOG-Treg cells and microglia in secondary lymphoid organs and CNS during the course of EAE. A**) Percentage of MOG-specific T_eff_ (red) or T_reg_ (blue) in the spleen after immunization. The upper graph shows the mean of the measurements from 3 animals each day for both populations (T_eff_ and T_reg_) together. The bottom panels show the distribution of all measurements (raw data) from 3 animals per day for each subpopulation as dots, with the mean in black; **B**) percentage of MOG-specific T_eff_ (red) or T_reg_ (blue) in the CNS from day 9 after immunization. The upper graph shows the mean of the measurements from 3 animals each day, while the bottom panels show the distribution of all measurements for each subpopulation as dots, with the mean in black; **C**) Clinical score of animals suffering EAE after immunization (mean + SD); **D**) Percentage of activated microglia in the CNS from day 9 after immunization.

In order to assess the presence of oscillation in T cell populations, their relationship and their behavior during the autoimmune response, we measured the frequency of both total and active MOG-specific T_eff_ and T_reg_ cell populations (compared with the non-specific T cells and the overall CD4+ cell population) using MOG-specific MHC class II tetramers during the course of EAE in the C57BL/6 mice immunized with MOG (Figure 1). T cells were measured in the spleen and CNS daily in series of animals (3 animals per day) immunized at the same time. As predicted by the cross-regulation model of T cell dynamics [10-12], we observed that the activation of MOG-specific T_eff_ and T_reg_ cells in peripheral lymphoid organs (spleen) follows an oscillatory behavior (Figure 2A). During the 30 days after immunization, we observed a consistent average time between peaks (period of oscillation) at around 4–5 days (Table 1), with coordinated dynamics between the two T-cell populations. Also, the oscillation amplitude (maximum percentage of antigen-specific cells with respect to total CD4+ cells) of MOG-specific T_eff_ cells was always significantly larger in average (between 2 and 6 fold) than the one for MOG-specific T_reg_ cells (Table 1). Additionally, the amplitude was always larger for the first two peaks, coinciding with the onset of clinical disease, and was attenuated along the course of the disease. We observed that both T cell populations were phase-locked, with differences between the time peaks being always smaller than 1 day, which indicates that the oscillations of T_eff_ and T_reg_ are tightly controlled by common mechanisms. In summary, after immunization, antigen-specific T_eff_ and T_reg_ populations display an oscillatory behavior. The robustness of the period observed also suggests the presence of a common regulatory mechanism, supporting the cross-regulation model.

**Table 1:** Statistics of period and amplitude of the oscillation of MOG-specific T cells and microglia in EAE

			**Period (days)**	**Amplitude (%)**				**Period (days)**	**Amplitude (%)**
**EAE**	**Spleen**	**MOG-T**_**eff**_	5 ± 0.7	0.8 ± 0.2 *	**EAE anti-CD20 therapy**	**Spleen**	**MOG-T**_**eff**_	6.7 ± 0.7	0.3 ± 0.1 *
		**MOG-T**_**reg**_	4.3 ± 0.9	0.14 ± 0.02 *			**MOG-T**_**reg**_	6.7 ± 2.7	0.06 ± 0.02 *
	**CNS**	**MOG-T**_**eff**_	4 ± 1	3.3 ± 0.9		**CNS**	**MOG-T**_**eff**_	3.0 ± 0.4	2.4 ± 1.4
		**MOG-T**_**reg**_	2.3 ± 0.9	4.0 ± 0.9 ***			**MOG-T**_**reg**_	3.3 ± 0.7	0.5 ± 0.2 ***
		**Microglia (activated)**	8	60 ± 7			**Microglia (activated)**	--	57 ± 9
			**Period (days)**	**Amplitude (cell num)**				**Period (days)**	**Amplitude (cell num)**
**Model EAE**	**Spleen**	**MOG-T**_**eff**_	5.2 ± 0.2 *	2254 ± 128 ***	**Model EAE anti-CD20 therapy**	**Spleen**	**MOG-T**_**eff**_	6.5 ± 0.5 *	816 ± 18 ***
		**MOG-T**_**reg**_	4.9 ± 0.2	552 ± 26 ***			**MOG-T**_**reg**_	5.5 ± 0.4	429 ± 10 ***

Period and amplitude of the peaks of active MOG-T_eff_ , MOG-T_reg_ and activated microglia in secondary lymphoid organs (spleen) and the CNS in animals developing EAE (as described in Figure 2), in model simulations (as described in Figure 4) and animals with EAE and treated with anti-CD20 therapy (as described in Figure 6). Amplitudes of MOG-specific T cells and activated microglia are shown as percentages to the CD4^+^ population and total microglia population, respectively; amplitudes in model simulations are expressed as cell number values. Data is shown as the mean ± SEM, statistical significance are between EAE versus EAE anti-CD20 therapy; sig. (*) p < 0.05, (***) p < 0.0001 (paired T test).

We also measured the frequency of MOG-specific T_eff_ and T_reg_ cells in the CNS of animals suffering EAE from the time at which cells migrate to the brain (from day 9). Because activated T-cells migrate to the target tissue after T-cell activation, we predicted a similar oscillatory behavior in the CNS by infiltrating T cells unless the blood–brain barrier (BBB) was buffering these oscillations. Accordingly, we observed the presence of MOG-specific T_eff_ and T_reg_ cells in the CNS at various times, again showing a coupled oscillatory dynamics. Amplitudes of T-cell incursions were around 3% and 4% of the total population of non-specific T-cells for T_eff_ and T_reg_, respectively (Figure 2B, Table 1) and always significantly larger than the one observed in the spleen, indicating the accumulation of antigen-specific cells in the target tissue. This is in agreement with previous observations in the same animal model showing that T-cell invasion of the CNS starts 2–3 days after T-cell activation in the periphery (day 9–10 post-immunization) and extends along the course of the disease (Figure 2C) [18].

**Figure 3:**
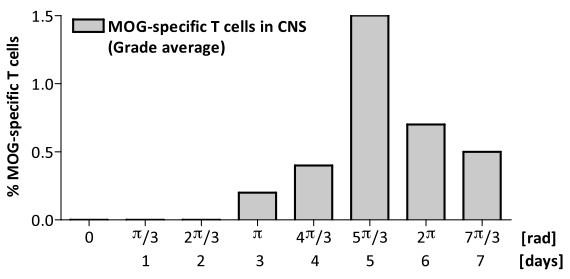
**Analysis of the time dependence between microglia activation and MOG-specific T-cell infiltration of the CNS.** Distribution of the average of incursion of MOG-specific T cells into the CNS (Teff and Treg) versus microglia phase cycle.

Considering that microglia are activated in response to CNS damage or by immunological mediators, we predicted a similar oscillatory behavior of activated microglia (CD45^low^CD11B^+^MHC-IA^b^) population following CNS invasion by MOG-T-cells. Accordingly, we observed the presence of oscillations in microglia activation after EAE induction (Figure 2D). Activated microglia shows two peaks of activation with a period of 8 days and a maximum amplitude of 60% (Table 1). Microglia remains activated for 6 days in both activation events, indicating an intrinsic activation period for microglia as well, and different for the one observed for T cells (Figure 2D). However, microglia activation started at day 10, preceding the peak of MOG-specific T cells within the CNS, and exhibits a first maximum at day 14, one day before the following peak of MOG- T_eff_ into the CNS. At same time, the maximum of clinical relapse was registered on day 13 to 14. The clinical score dropped from day 14 to day 21 (Figure 2C), coinciding with the microglia deactivation to baseline levels. The second microglia activation peak spans from days 20 to 26, again preceding the following peak of activated MOG- T_eff_ cells in the CNS (Figure 2B). Overall, our data indicates activation of microglia preceding or concomitant with the invasion of CNS by MOG specific T-cells. In order to quantify the dependence of microglia activation on CNS infiltration by MOG-specific T-cells, we plotted the distribution of phases within a microglia cycle for which the average of MOG-specific T-cell incursion is observed in the CNS, observing again that peaks of microglia activation precede the infiltration of MOG-specific T cell to the CNS (Figure 3). Together, these findings indicate that microglia activation also oscillates during brain autoimmunity, following MOG-specific T-cell activation in the periphery but preceding the incursion of those cells into the CNS. This result suggests the need for additional signals for microglia activation preceding antigen-specific T cell infiltration of the CNS and/or a role for microglia in the recruitment of MOG-specific T-cell within the CNS.

### Mathematical modeling identifies T_reg_ activation as a major element governing autoimmune susceptibility

**Figure 4:**
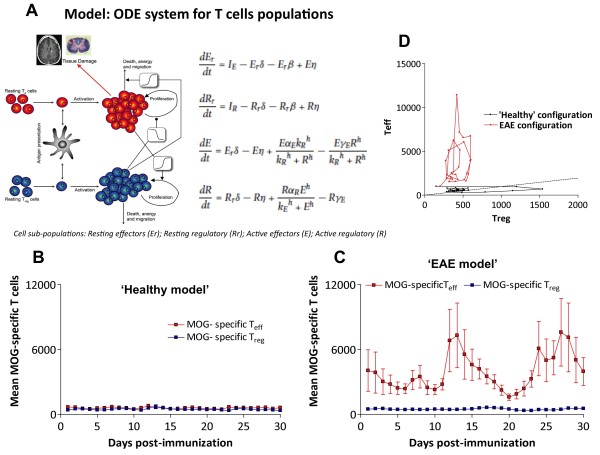
**Simulations from the cross-regulation T-cell model. A**) Graphical representation and the equations of the T-cell cross-regulation model as described in [10]. Parameters of the model are described in Additional file 1: Table S1. Simulation of the time course of the number of MOG-specific T_eff_ (red) or T_reg_ (blue) cells in the spleen (y-axis) after immunization (x-axis, in days) by the cross-regulation model in ‘healthy’ (**B**) and in ‘EAE’ configuration regimes (**C**). Simulations are shown by mean ± SD of 3 simulations for 30 days (discretized by days) to express average points similarly to experimental data in Figure 2A; **D**) Phase-space plot shows T_eff_/T_reg_ trajectories in simulations (30 days) for both configurations (‘healthy’ in black and ‘EAE’ in red).

In order to interpret the experimental data described above, we make use of a cross-regulation model of T-cell activation previously described (Figure 4A) [10] after adapting it to reproduce mice data (Additional file 1: Table S1). The model is based on the hypothesis of the strong influence of a negative feedback between T_eff_ (E) and T_reg_ (R) cells in the adaptive immune response, using a predator–prey system (see equations in Figure 4A). Active T_eff_ would produce signals to evoke expansion of T_reg_ population; meanwhile, the increase of T_reg_ activity would mean a suppressive effect on the levels of active T_eff_. The model also includes thymic resting/naïve T cells (R_r_ and E_r_, Figure 4A) arising from a discrete stochastic input of cells in the immune system, mimicking the apparently random nature of the pulse trains of resting cells (which can become activated by antigen presentation). We identified two key characteristics of the cross-regulation model that clearly account for the difference between the healthy and EAE behavior, namely the average amplitude of the peaks and the average periodicity. Using those features, we established that the rate of T_reg_ activation (α_reg_) and the number of T_eff_ cells required for half-maximal activation of T_reg_ cells (K_eff_) were found to be critical for reproducing the oscillatory autoimmune behavior data versus the healthy configuration parameters. In particular, using the same value for the rate of antigen presentation in both sub-populations (δ), we observed oscillatory behavior after a decrease of α_reg_ (down to 1.05 /day; range: 0.75 to 1.05) and an increase of K_eff_ (up to 2,500 cells) (Figure 4 B, C). Thus our data and model sensitivity support the concept that the observed oscillatory autoimmune response could be explained by a decrease in the strength of the T_eff_-T_reg_ cross-regulation, a combination of increasing the T_eff_ threshold for activating T_reg_ cells and reducing the number of activated T_reg_ cells.

In agreement with our experimental data, the mathematical simulations showed that the coordinated oscillations of the active populations of T_eff_ and T_reg_ cells have amplitudes for T_eff_ at least 4 times larger in average than T_reg_ (Table 1). Also, in order to compare the adapted model to mice conditions with our experimental data, we tested the average distribution of standard deviations against the means of the oscillation amplitudes for three simulations (1 point per day) for 30 days. Means of relative deviations measured in the experimental data was 0.62 ± 0.01 for T_eff_ cells and 0.40 ± 0.02 for T_reg_ cells, while in model simulations it was 0.61 ± 0.01 for T_eff_ and 0.30 ± 0.01 for T_reg_ cells. These results fit qualitatively with the experimental observations in the EAE dataset.

The model proposes a predator–prey system in which T_reg_ and T_eff_ play the active-subsets of predator and prey populations, respectively. We performed a phase-space representation of the mathematical model trajectories in the plane for T_eff_-T_reg_ after fitting to experimental EAE data (Figure 4D), showing that the healthy-state dynamics are controlled by T_reg_ (x-axis), while the autoimmune-state dynamics are mainly governed by T_eff_ (y-axis). In summary, the combination of experimental data with theoretical modeling supports the importance of cross-regulation of T-cell populations in the control of the immune response, and points to T_reg_ cells as a critical population in order to understand dynamics of autoimmune diseases.

### B cell depletion modulates T-cell subpopulation dynamics differentially, influencing the outcome of EAE

**Figure 5:**
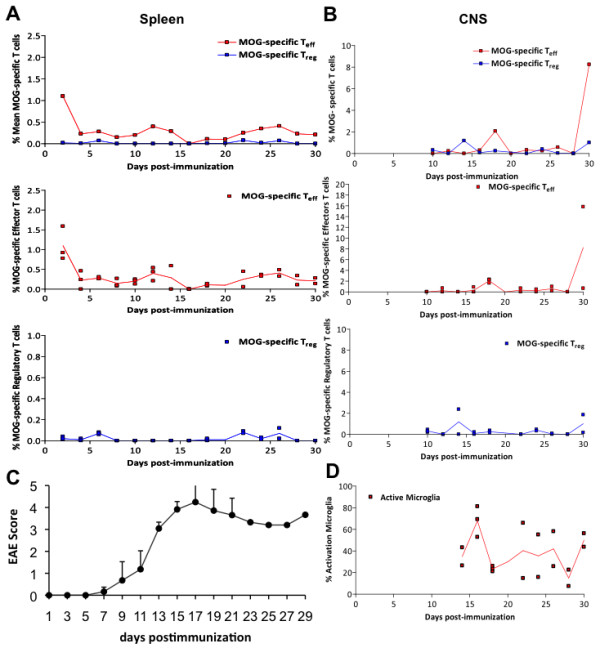
**Dynamics of activated MOG-T**_**eff**_**, MOG-T**_**reg **_**cells and microglia in secondary lymphoid organs and CNS during the course of EAE after B cell depletion. A**) Percentage of MOG-specific T_eff_ (red) or T_reg_ (blue) cells in the spleen after immunization. The upper graph shows the mean of measurements from 3 animals each day, and the bottom panels show the distribution of all measurements for each subpopulation as dots, with the mean in black; **B**) percentage of MOG-specific T_eff_ (red) or T_reg_ (blue) cells in the CNS from day 9 after immunization. The upper graph shows the mean of measurements from 3 animals each day, and the bottom panels show the distribution of all measurements for each subpopulation as dots, with the mean in black; **C**) clinical score of animals suffering EAE after B cell depletion (mean + SD); **D**) percentage of activated microglia in the CNS from day 9 after immunization.

Recently, it has been shown that B-cell depletion by anti-CD20 antibodies has a strong effect in suppressing the relapsing-remitting course of autoimmune diseases such as RA and MS [13,14], modulating the frequency of T-cell subpopulations [15]. In order to analyze the influence of B-cells on T-cell dynamics, we analyzed the frequency of antigen-specific T_eff_ and T_reg_ subpopulations in mice during the course of EAE after B-cell depletion. We made use of C57BL/6 mice immunized with MOG peptide, given that in this model B-cells are not encephalitogenic, but rather have been shown to play a role in T cell activation [17]. As expected, we observed that anti-CD20 therapy induced a rapid, almost complete depletion of B-cells in the peripheral lymphoid organs (Additional file 1: Figure S1). Regarding the dynamics of T-cells, we observed that after B-cell depletion, MOG-specific T_eff_ and T_reg_ subpopulations maintained their oscillatory presence in secondary lymphoid organs, although in this case the large activation peaks of T_eff_ that appeared in non-treated animals suffering EAE were significantly decreased in amplitude and appeared at earlier times (Figure 5A). However, similarly to untreated animals suffering EAE, the peaks of T_eff_ and T_reg_ were phase-locked. Interestingly, in the CNS T_eff_ and T_reg_ incursions were present as well, but with a significant decrease in the amplitude of T_reg_ cells compared to T_eff_ cells (Figure 5B, Table 1). Surprisingly, even if the frequency of both MOG T-cell subpopulations were decreased, microglia were activated to a similar extent as in untreated animals with EAE (~60% in the first peak with a maximum at day 16), followed by oscillations without a clear period or phase, and did not fall to baseline levels (Figure 5D), suggesting a transition to chronic activation and contributing to the worsening of the disease. At the same time, the maximum clinical score was delayed compared with untreated animals with EAE (day 17 in Figure 5C compared to day 12 in Figure 2C) and disease was more severe, as previously described in this model [17]. Both groups showed statistically significant differences by paired-t test (with p < 0.05), specifically at days 11 (early phase of the disease episode), 17 (maximum score of treated EAE group, Figure 5C), at 21 (late phase of the disease episode).

The presence of an imbalance in the CNS of the peaks of antigen-specific T_eff_ compared to T_reg_ and the persistence of microglia activation may account for the impairment of EAE in this model despite anti-CD20 therapy. Therefore, our results suggest that B-cell depletion modulates T-cell activation in peripheral immune organs with more profound effects in T_reg_ activation (Table 1, *, p < 0.05). Downregulation of T_reg_ was mainly revealed in the CNS tissue (Table 1, ***, p < 0.0001), and was associated with persistent microglia activation and (as mentioned before) with the higher clinical severity in the pre-treated EAE group. However, the fact that the oscillatory dynamics of MOG-specific T-cells remain suggests that B-cell depletion works by tuning T-cell activation thresholds, rather than reverting the autoimmune process itself.

### Sensitivity analysis of the cross-regulation model support the critical role of B-cell depletion on T_reg_ activity

**Figure 6:**
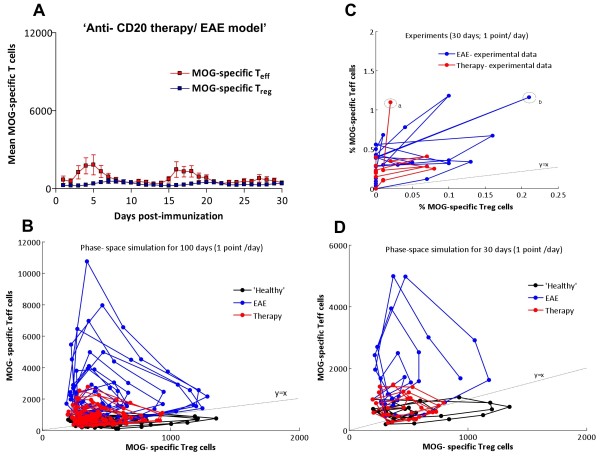
**Modeling the effect of anti-CD20 mediate B-cell depletion in the cross-regulation T-cell model. A**) Simulation of the time course of the number of MOG -specific T_eff_ (red) and Tr_eg_ (blue) cells in the spleen (y-axis) after immunization (x-axis, in days) by the cross-regulation model using values for α_reg_ and K_eff_ from the ‘EAE’ regime (see Results section). **B**) Phase-space plots showing T_eff_/T_reg_ cell counts trajectories in simulations of 100 (B) and 30 (D) days for ‘healthy’ (black), EAE (blue) and B-cell depletion (red) simulations. The 30-day simulation (**D**) was performed in order to compare with experimental data in **C** (in C, points ‘a’ and ‘b’ are marked as first peaks recorded in experiments for both groups compared). Simulations were discretized in days to show data points similar to experimental data in Figure 5A.

In order to analyze the influence of B-cell depletion on the dynamics of T-cell subpopulations, we performed a sensitivity analysis of our mathematical cross-regulation model with the aim of identifying the model parameters governing the dynamics induced by this therapy in T cells. Although our model does not include B- cells, by fitting the model with the experimental data we were able to analyze the resulting dynamics of both T-cell populations after anti-CD20 therapy and obtain insights into a possible mechanism for explaining how B-cells influence T-cell dynamics. We found that the dynamics of the antigen-specific T-cell subpopulation after anti-CD20 therapy was reproduced by reducing the K_eff_ threshold below the healthy standard (<1,000 cells; e.g. 850 cells), independently of the α_reg_ parameter (α_reg_ values were in the low range of autoimmune regime; Figure 6). In other words, our mathematical model suggests that B-cell depletion therapy may influence the autoimmune process by preventing uncontrolled activation of T_eff_ without strengthening T_reg_ activation. Experimental trajectories through the phase-space were dominated by the T_eff_ cells (y-axis, Figure 6C) in EAE (in blue) and in anti-CD20 therapy (in red). However, in the anti-CD20 treated group, we did not observe the high excursions characteristic of EAE. This behavior can be easily modeled by performing simulations for 30 days, which highlights the fact that B cell depletion, although suppressed the T_eff_ excursions, did not recover the T_reg_ excursions observed in the healthy cohort (Figure 6D).

We performed simulations of the changes in the survival/death rate of T cell populations in order to predict its effects in the oscillatory dynamics of T cells. We observed that a 2-fold decrease or increase in the cell death rate of T_eff_ or T_reg_ decreases the period by at least one day for active T_eff_ cells and two days for T_reg_. Although peak period (period of time between two consecutive maximums of activation) is not a well-known characteristic of T cell activation, it would be a good marker to confirm the oscillatory nature of this process. In summary, our simulations suggest that B-cell depletion modulates autoimmune diseases mainly by affecting the interaction between T_reg_ and T_eff_, and in particular strengthening the T_eff_-T_reg_ cross-regulation by lowering the T_reg_ activation threshold.

## Discussion

In this study we have assessed the dynamics of antigen-specific T-cell subpopulations after immunization and related them to the outcome of brain autoimmune disease. We observed that, after immunization, antigen-specific effector and regulatory T-cells are activated and oscillate in a coupled manner with an intrinsic period. The combination of experimental and computational analysis supports the concept that relapses in autoimmune diseases are an intrinsic property of the regulation of the immune system, that can be modulated by environmental factors [10]. The fact that oscillations of both populations were matched day-to-day (phase-locked), indicates the importance of the synchronous activation of effector and regulatory adaptive immune response for the outcome of the immune response [19]. Previous models of T-cell activation suggest that regulatory T-cell activation follows effector T-cell activation with some delay [20-22]. However, the fact that our experimental data shows that both regulatory and effector T-cells become activated the same day after immunization points the importance of triggering the regulatory T-cell response at the same time and in parallel than the effector response. After activation, the negative feedback between both populations could maintain their oscillation for long period of time, although removal of the antigens and other suppressive factors cancels the oscillations in the long term. The analysis of the T cell cross-regulation model after fitting with the experimental data supports a key role of regulatory T-cell dynamics in defining the outcome of the autoimmune response [22,23]. Our model could be further validated by future experimental studies testing, for example, different scenarios of T-cell viability assays (involving e.g. cellular pathways implicated in the control of cell death and survival, such as Jak/Stat, Ras/MAPK, PI3K/AKT, and interleukin signaling).

During the course of EAE, activated CD4+ and CD8+ cells enter the brain, which is associated with microglia activation that mediates demyelination and axonal loss [18]. In the EAE model, regulatory T-cells have a critical role in controlling the activation of encephalitogenic T-cells, but their mechanism of action is complex. Regulatory T cells are dependent on the local (CNS) conditions that regulate their activation and suppressive activity [24,25]. However, the functional outcome seems to be dependent on balancing the suppression of the activity and proliferation of effector T-cells with the promotion of the survival and activity of encephalitogenic T-cells, causing their accumulation and distorting the regulatory and effector T-cell balance [26].

Another interesting observation from our results is that the activation of the innate immune system, namely microglia activation, also follows the oscillatory dynamics of the adaptive immune response with a well-defined phase. Interestingly, we observed that microglia become activated closely after activation of T-cells in the periphery, but before the infiltration of antigen-specific T-cells into the CNS. This results points to a model in which the autoimmune response organized in secondary lymphoid organs or in the circumventricular organs of the brain delivers signals (most likely soluble factors) to the brain parenchyma, activating microglia. Time series analysis of gene expression changes along the course of EAE have shown activation of genes related with the innate immune response before the presence of inflammatory infiltrates, supporting the role of microglia activation in the early stages of the disease, even preceding an antigen-specific T-cell invasion of the CNS [27]. Recent pathological studies in the brain of patients with MS as well in EAE models suggest that activated effector T-cells accumulate in subaracnoid space where they can be restimulated, releasing pro-inflammatory signals that activates microglia and contributes to the opening of the blood–brain barrier and subsequent infiltration into the brain by T-cells of the CNS parenchyma [28-30].

Amplitudes of MOG T-cell activation peaks were clearly larger in the early stages of the disease following immunization and then decreased. This could be explained by several factors. After immunization, the availability of antigens at site of immunization to be presented to T-cells by antigen presenting cells is significantly higher compared with the antigen concentration within the CNS in consecutive restimulations. Also, immunization exerts a synchronization effect that makes it easier to detect the activation of T-cells right after the animal is immunized, in comparison with later times at which individual clones become increasingly desynchronized, complicating the detection of T-cell expansion. Another factor may be the effect of epitope spreading, which involves the recruitment of other antigen specificities that were not measured here. Also, anti-inflammatory signals from the tissue, such as IL-10 would contribute to shutdown the immune response and therefore dampening of the peaks of activated T-cells.

B-cells seem to have a complex role in brain autoimmunity. In addition to their role as antigen producing cells, B-cells have been shown to contribute to the pathogenesis of autoimmune disease via the production of a pro-inflammatory factors such as IL-6, the activation of pro-inflammatory Th1 and Th17 cells and monocytes, and the inhibition of T-cell activation by regulatory B (B10) cells [15,17,31-34]. The role of B-cell depletion in the activation of antigen specific T-cells in EAE is complex as B-cells do not directly impair T-cell function, but rather alter the balance between effector and regulatory function of these cells [35-37]. Our results indicate that in a model in which anti-CD20 therapy induces a more severe disease course, even if B-cell depletion decreases the activation of both effector and regulatory T cells, the disease outcome seems to be dependent, at least in part, on the balance of these two populations. We show that the activation of MOG-specific T-cells happens earlier and faster in anti-CD20 treated animals than in non- B-cell depleted animals. This is agreement with the view that effector B-cells modulate antigen presentation to T cells and regulatory B-cells modulate T cell expansion [37,38]. In the CNS of B-cell depleted animals, we observed a more significant effect on regulatory than effector response and a subsequent enhancement of microglia activation, which correlates with more severe CNS tissue damage.

Our results have several implications for immunotherapy. First, immunomodulatory therapies, such as anti-CD20 therapy, do not revert the pathogenic response in autoimmune diseases, but rather maintain them in a dynamic state that is less deleterious. For this reason, cessation of the therapy would allow to the immune system to come back to its original autoimmune dynamics and in some cases, even worsen the disease due to a rebound effect. This has been well documented in cessation of natalizumab therapy in humans, in which a significant proportion of patients develop a rebound relapse a few months after the last natalizumab infusion. Second, although regulatory T-cells are an ideal target for immunotherapy – e.g. increasing their function for treating autoimmune disease or decreasing their function for cancer immunotherapy - the outcome of the disease will be dependent on the dynamics of all populations implicated and the timing in which the therapy is started. For this reason, the ideal therapy at this level would involve fine-tuning the long-term dynamics of antigen-specific T-cells to maintain them at a level closer to the healthy state.

Our study has several limitations. First, we have quantified antigen-specific T-cells using MHC class II tetramers by flow cytometry, which has a resolution for identifying 1 in every 10^4^ target cells in the tissue from a standard acquisition of 2–5 x 10^5^ leukocytes [39]. Moreover, because we attempted to quantify MOG specific T cells within the context of the natural repertoire of a rodent, instead of using transgenic animals with single TCR specific for MOG (which may have simplified the identification of MOG specific T-cells), quantification was done in different animals and individual dynamics were extrapolated from the population analysis. For this reason, we analyzed results as proportions to the reference CD4+ cell population instead using absolute numbers. While the presence of noise created by technical limitations might have prevented from observing more cycles due to desynchronization of the T-cell activation between animals, the oscillations reported here were robust enough to be captured with this technique. Also, we focused in the immune response specific to the single antigen used for immunization (MOG), but did not assess the role of epitope spreading in the dynamics of autoimmune T cells nor in the dynamics of other lymphocyte subpopulations (e.g. CD8+ cells) with other specificities, which may have a role in the outcome of the autoimmune response. Finally, anti-CD20 produces profound but not complete B-cell depletion, and does not target only MOG-specific B-cells. For this reason, residual B-cells may have played a role in the worsening of EAE. Alternatively, anti-CD20 therapy may preferentially target some B-cell subpopulations such as IL-10- or IL-6-producing B cells, or cells at different stage of differentiation that may have a specific effect on T-cell dynamics. However, the results from our study support previous models of the regulation of T- cell populations and their role in the immune response.

## Conclusion

Our study supports the importance of the cross-regulation between lymphocyte populations and their contribution to the outcome of the immune response. Moreover, highlights the role of regulatory T-cells in driving the dynamics of the immune response. Even if these cells are a smaller population compared with naive and effector cells, their critical role in the regulation of immune response makes them central in the pathogenesis of autoimmune diseases, and thus attractive targets for immunotherapy against these diseases, even in an indirect way such as is the case in anti-CD20 therapy.

## Methods

### Animals and induction of EAE

EAE was induced in C57BL/6 (I-A^b^) mice by injecting them with an emulsion containing 300 μg of MOG_35-55_ peptide and 500 μg of *M. Tuberculosis* extract in incomplete Freund adjuvant subcutaneously into the flanks as described before [40]. Mice receive 0.2 ml of the emulsion in the flank. In addition, the mice receive 500 ng of *B. Pertussis* toxin via intraperitoneal injection (i.p) in 200 μl PBS on days 0 and 2. Clinical signs of EAE were assessed according to the following score: 0, no signs of disease; 0.5, partial loss of the tone in the tail; 1, loss of tone in the tail; 2, hind limb paresis; 3, hind limb paralysis; 4, tetraparesia; 5, tetraplegia; 6, moribund [6]. Moribund mice were given disease severity scores of 6 and euthanized. For each experiment, we made use of 3 animals per day (or every other day for repetitions) for 30 days, and the experiments were repeated twice. The study was approved by the ethical committee on animal research of the University of Barcelona.

### Tissue preparation and T-cell isolation

Splenocytes were obtained from the spleen by digesting it with collagenase D (Roche) and Dnase I (Roche) at 37°C for 45 min. Mononuclear cells were isolated by passing the tissue through a cell strainer (70 μm) followed by a Ficoll (Sigma) gradient centrifugation. T cells from the CNS were obtained by collecting the forebrain, cerebellum and spinal cord. CNS tissue was cut into small pieces and digested with collagenase D (Roche) and Dnase I (Roche) at 37° C for 45 min. Mononuclear cells were isolated by passing the tissue through a cell strainer (70 μm) to obtain single cell suspensions. Leukocytes were isolated from the CNS by gradient centrifugation. Briefly, a Percoll (Sigma) gradient (70/37%) centrifugation was made and inter-phase between 70% and 37% phase was taken. Myelin in the upper layer was removed. Cells harvested from the gradient inter-phase and the upper-phase was washed in PBS and resuspended.

### Tetramers purification and cell staining

MOG_35-55_/IA^b^ tetramer construct was generously provided by Prof. Vijay Kuchroo, from Harvard University, and purified as previously described [25]. Tetramers were incubated with PBS, 0.2% BSA, 0,1% sodium azide for three hours at 37°C at darkness. After washing, cells were stained with 7-AAD, (BD Pharmingen) and antibodies against CD4 (BD Pharmingen), CD62L (BD Pharmingen), CD25 (BD Pharmingen), CD69 (BD Pharmingen), and CD45 (BD Pharmingen). For microglia activation, cell were stained with anti-MHC class II (IA^b^) (Abcam), CD11b (BD Pharmingen) and CD45 (BD Pharmingen). B-cell staining was performed using anti CD45R/B220 (BD Pharmingen) and anti-CD21 (BD Pharmingen) antibodies. Stained cells were analyzed on a FACSCanto machine (BD biosciences) and data analysis was performed with FACS Diva software.

### Lymphocyte and microglia subpopulations analysis

Antigen specific T cells were characterized by being tetramer positive (IA^b^-_MOG_+). MOG-specific T_eff_ cells were gated as the CD45^+^CD4^+^CD25^-^CD69^+^IA^b^-_MOG_^+^ population [25,41-43] (Figure 1A). MOG-specific T_reg_ cells were gated as the CD45^+^CD4^+^CD25^hi^IA^b^-_MOG_^+^ population [8,44,45] (Figure 1B). We did not check Foxp3 expression on the T_reg_ population because it requires cellular permeabilization, which was not compatible with the tetramer staining. Nevertheless, the subset analyzed corresponds to T_reg_ population as described before [25]. Also, we analyzed the expression of CD69 and CD62 since there is a subpopulation of T_reg_ cells that expressed CD69. Non-encephalitogenic T_eff_ lymphocytes were characterized by being CD45^+^CD4^+^CD25^-^IA^b^-_MOG_^-^ (Figure 1C). Non-encephalitogenic T_reg_ lymphocytes were characterized by being CD45^+^CD4^+^CD25^hi^IA^b^-_MOG_^-^ (Figure 1D). CD69 and CD62 expression was also analyzed in non-encephalitogenic T-cells. B-cells were analyzed after staining with B220 and CD21 antibodies [31,46]. Depletion of mature B-cells (B220^+^CD21^+^) was examined in the spleen and CNS every day over the disease course in the group of mice that received anti-CD20 treatment [17]. Activated microglia was defined as the CD45^low^CD11B^+^MHC-II-IA^b+^ population (Figure 1C) [47,48]. MOG-specific T cells and non-encephalitogenic T cells (either T_eff_ or T_reg_) are shown as percentages with respect to the CD4^+^ population (MOG-Teff /CD4^+^; MOG-Treg/CD4^+^; Teff/CD4^+^; Treg /CD4^+^) in order to adjust for differences in total cell count per day. B lymphocyte results are shown as percentages of total lymphocytes (B Lymphocytes/Total Lymphocytes). Microglia activation is shown as the IA^b^ percentage of total microglia.

### Anti-CD20 therapy

Mouse anti-CD20 monoclonal antibody was generously provided by Andrew Chan from Genentech. To deplete B-lymphocytes in vivo, animals received anti-CD20 antibody 200 μg in 250 μl PBS one week before immunization as described before [17,31].

### Mathematical model, simulations and sensitivity analysis

We make use of a model of active T_eff_-T_reg_ cross-regulation developed previously to describe the dynamics of T-cells in humans [10], updating here the model parameters to reproduce experimental data from EAE studies in mice. The model is based in 4 differential equations describing the dynamics of antigen specific resting T_eff_ (1), resting T_reg_ (2), activated T_eff_ (3), and activated T_reg_ (4).

(1) $$\frac{dE_{r}}{\text{dt}}=I_{E}-E_{r}\delta -E_{r}\beta +E\eta$$

(2) $$\frac{dR_{r}}{\mathrm{dt}}=I_{R}-R_{r}\delta -R_{r}\beta +\mathrm{R\eta}$$

(3) $$\frac{\mathrm{dE}}{\mathrm{dt}}=E_{r}\delta -\mathrm{E\eta}+\frac{E\alpha_{E}k_{R}{}^{h}}{k_{R}{}^{h}+R^{h}}-\frac{E\gamma_{E}R^{h}}{k_{R}{}^{h}+R^{h}}$$

(4) $$\frac{\mathrm{dR}}{\mathrm{dt}}=R_{r}\delta -\mathrm{R\eta}+\frac{R\alpha_{R}E^{h}}{k_{E}{}^{h}+E^{h}}-R\gamma_{E}$$

Specifically, parameter changes leading to time rescaling were performed in order to adapt the characteristic period of T-cell oscillations from the values observed experimentally, with respect to previous theoretical values for humans [10] (Additional file 1: Table S1). In this new version of the model we also represent the pulse train input of naive T-cells from the thymus by a noise-driven excitable dynamics typical of some gene circuit architectures, as a mathematical tool to mimic continuous random activity pulses [49]. We simulated all stochastic differential equations of the model by means of a stochastic Runge–Kutta method of second order (Heun method) using Matlab software.

We performed a sensitivity analysis of the model by increasing and decreasing the baseline parameters (Additional file 1: Table S1) by 20% and 50%, and collecting the statistics of the average amplitude and period of activated T_eff_ and T_reg_ populations. This sensitivity analysis was performed by running simulations over a time of 300 days in model units using the Matlab software. Simulations are initiated with no resting T_eff_ and T_reg_ cells, and 1,000 and 200 activated T_eff_ and T_reg_ cells, respectively, as described before [10].

### Statistical analysis

All experiments were performed at least two times. The values are expressed as the means ± SEM. We use the Mann-Withney test to determine statistical significance between groups and Wilcoxon signed-rank test for differences in time series. Statistical analyses were performed using PASW 18.0 software (IBM) and GraphPad Prism 4 software.

## Abbreviations

MS: Multiple Sclerosis; EAE: Experimental Autoimmune Encephalomyelitis; Teff: Effector T cells; Treg: Regulatory T cells; MOG: Myelin olygodendrocyte glycoprotein; MRI: Magnetic resonance imaging; RA: Rheumatoid Arthritis.

## Competing interest

This work was supported in part by an unrestricted grant from Roche. PV has received consultancy fees from Roche as part of scientific advice regarding the mechanism of action of anti-CD20 antibodies for the treatment of MS. All the other authors have no competing interest.

## Authors’ contributions

SMP and BM performed animal model and immunological studies and analyzed data; EA and NV developed the ODE model and performed simulations and sensitivity analysis; JGO, IMF and PV: designed the study, supervised the study and wrote the paper. All authors read and approved the final manuscript.

## Supplementary Material

Additional file 1 Table S1 Parameters of the model and initial conditions. **Figure S1.** B cell counts after anti-CD20 therapy. Percentage of B cells in the spleen (L-B spleen) and the CNS (L-B CNS) from two immunized animal per day are plotted along the duration of the experiment (30 days). **Figure S2.** Predictions of the effects of changes in the survival/death rate of T cell populations in the oscillatory dynamics of T cells. Graphs shows simulations of the effect of a 2-fold decrease in the cell death rate of T_eff_ and T_reg_ in the period of T cell population oscillations. Click here for file
