# Supplementary material for: Dynamic cross-regulation of antigen-specific effector and regulatory T cell subpopulations and microglia in brain autoimmunity
Source: BMC Syst Biol. 2013 Apr 26;7:34. doi: 10.1186/1752-0509-7-34 (PMC3651362; doi:10.1186/1752-0509-7-34)
Supplement: Additional file 1 Table S1 — Parameters of the model and initial conditions. Figure S1. B cell counts after anti-CD20 therapy. Percentage of B cells in the spleen (L-B spleen) and the CNS (L-B CNS) from two immunized animal per day are plotted along the duration of the experiment (30 days). Figure S2. Predictions of the effects of changes in the survival/death rate of T cell populations in the oscillatory dynamics of T cells. Graphs shows simulations of the effect of a 2-fold decrease in the cell death rate of Teff and Treg in the period of T cell population oscillations. [file 1752-0509-7-34-S1.docx]

**Supplementary material**

**Supplementary Table S1. Parameters of the model and initial conditions**

| **Initial Conditions** | **Symbol** | **Values (Mice)** | **Values (Human)** |
| --- | --- | --- | --- |
| Resting Teff cell population size | *E_r_* | E_n0_= 0 cells | E_n0_= 0 cells |
| Resting Treg cell population size | *R_r_* | R_n0_= 0 cells | R_n0_= 0 cells |
| Activated Teff cell population size | *E* | E_a0_= 1,000 cells | E_a0_= 1,000 cells |
| Activated Treg cell population size | *R* | R_a0_= 200 cells | R_a0_= 200 cells |
| **Parameter description** | **Symbol** | **Values** | **Values** |
| Antigen Presentation | δ | 3 day^-1^ | 1 day^-1^ |
| Anergy | β | 0.03 day^-1^ | 0.01day^-1^ |
| Memory | η | 0.03 day^-1^ | 0.01 day^-1^ |
| Maximum Teff proliferation rate | α_eff_ | [3:6] day^-1^ | [1:3] day^-1^ |
| Maximum Treg proliferation and activation rate | α_reg_ | [0.75:6] day^-1^ | [0.25:2] day^-1^ |
| Teff death, anergy and migration Rate | γ_eff_ | 0.6 day^-1^ | 0.2 day^-1^ |
| Treg death, anergy and migration Rate | γ_eff_ | 0.6 day^-1^ | 0.2 day^-1^ |
| Teff cell population sizes leading to half maximal effect counterpart | *K_eff_* | 1,000 cells | 1,000 cells |
| Treg cell population sizes leading to half maximal effect | *K_reg_* | 200 cells | 200 cells |
| Hill coefficient | *h* | 5 | 5 |

**Supplementary Figure S1. B cell counts after anti-CD20 therapy.** Percentage of B cells in the spleen (L-B spleen) and the CNS (L-B CNS) from two immunized animal per day are plotted along the duration of the experiment (30 days).

**
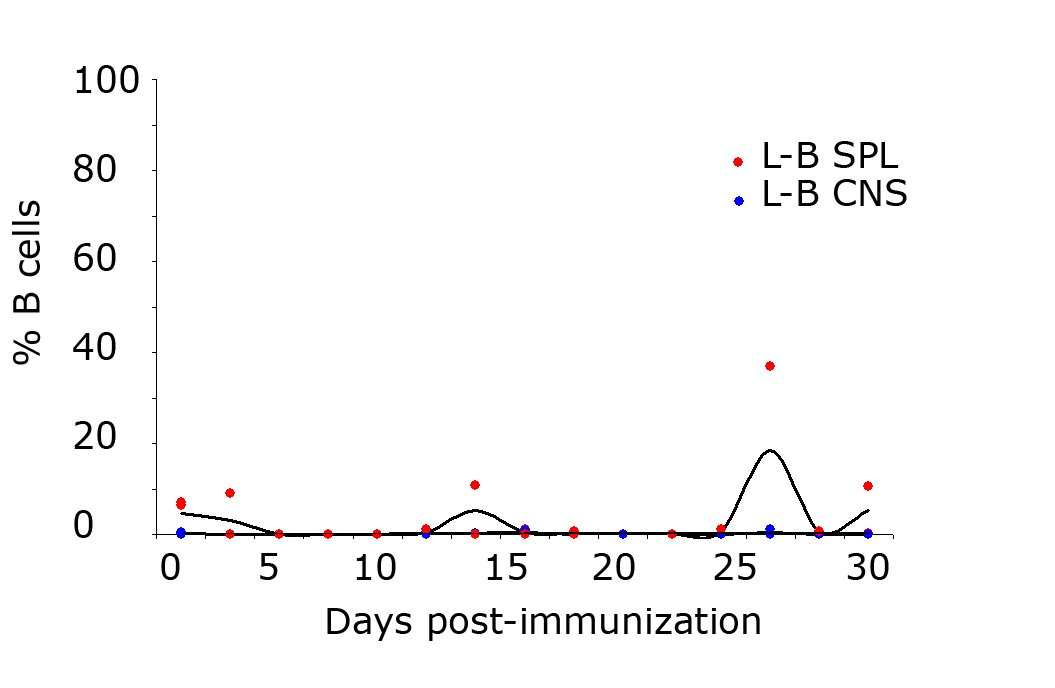
**

**Supplementary Figure S2. Predictions of the effects of changes in the survival/death rate of T cell populations in the oscillatory dynamics of T cells.** Graphs shows simulations of the effect of a 2-fold decrease in the cell death rate of T_eff_ and T_reg_ in the period of T cell population oscillations.

**
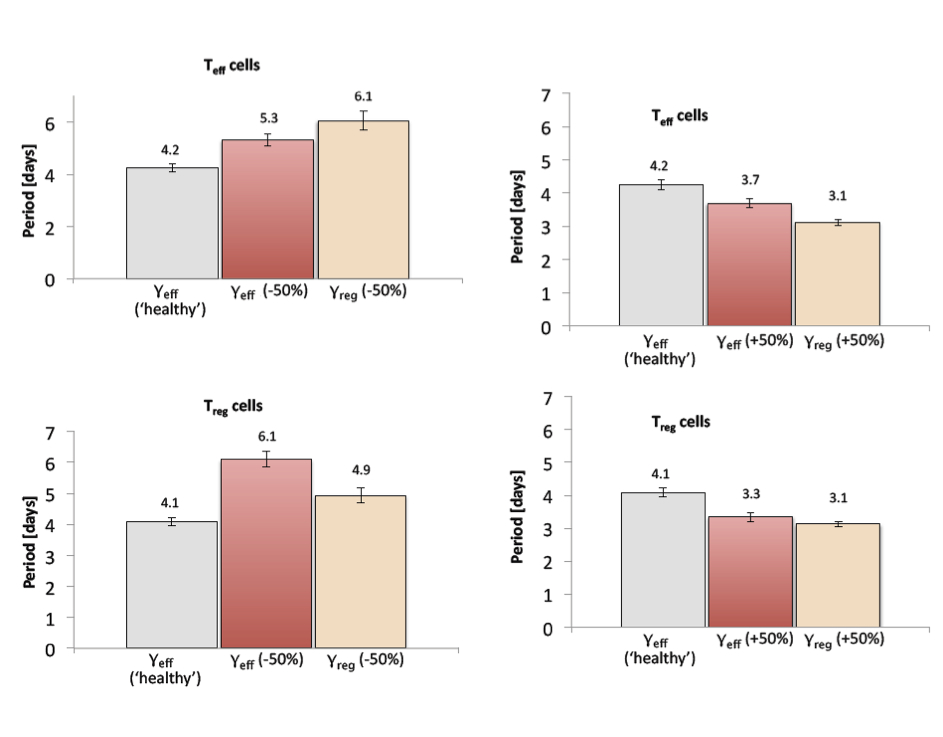
**
